# Breaking the Limits: Transcarotid Valve-in-Valve Transcatheter Aortic Valve Implantation With Bioprosthetic Valve Fracture in a Small Mitroflow and Extracorporeal Membrane Oxygenation Rescue

**DOI:** 10.1093/icvts/ivaf298

**Published:** 2026-01-20

**Authors:** Víctor X Mosquera, José M Martinez-Comendador, José J Cuenca-Castillo

**Affiliations:** Department of Cardiac Surgery, Complejo Hospitalario Universitario de A Coruña, A Coruña 15006, Spain; Instituto de Investigación Biomédica de A Coruña (INIBIC), A Coruña 15006, Spain; Department of Cardiac Surgery, Complejo Hospitalario Universitario de A Coruña, A Coruña 15006, Spain; Department of Cardiac Surgery, Complejo Hospitalario Universitario de A Coruña, A Coruña 15006, Spain; Instituto de Investigación Biomédica de A Coruña (INIBIC), A Coruña 15006, Spain

**Keywords:** TAVI, Valve-in-valve, VA ECMO, bioprosthesis cracking, Mitroflow bioprosthesis

## Abstract

**Background:** Valve-in-valve transcatheter aortic valve implantation (ViV TAVI) in small surgical bioprostheses presents unique challenges due to high residual gradients and risk of coronary obstruction.

**Case summary:** We report a case of an 86-year-old man with a degenerated Mitroflow 19 mm valve who underwent ViV TAVI via transcarotid access using a 20 mm Myval valve. Significant underexpansion was observed post-implantation, with elevated transvalvular gradients. Bioprosthetic valve fracture (BVF) was performed using an 18 mm non-compliant balloon, resulting in full valve expansion and gradient reduction. Shortly after BVF, the patient developed myocardial stunning and hemodynamic collapse, requiring urgent veno-arterial extracorporeal membrane oxygenation (VA ECMO) support. Extracorporeal membrane oxygenation was successfully weaned after 48 hours, and the patient was discharged in stable condition.

**Conclusion:** This is the first reported case of transcarotid ViV TAVI with BVF in a 19 mm Mitroflow bioprosthesis, successfully rescued with ECMO. The report highlights the importance of appropriate access planning, valve selection, and ECMO standby in complex high-risk anatomies.

## INTRODUCTION

Valve-in-valve (ViV) transcatheter aortic valve implantation (TAVI) is a valuable alternative to re-do surgery in patients with structural valve degeneration. Small surgical valves, such as the Mitroflow 19 mm, pose technical and haemodynamic challenges due to limited true internal diameter and high risk of coronary obstruction. Bioprosthetic valve fracture (BVF) can improve expansion and gradients but is rarely performed via non-femoral access.^1–3^

We report a unique case of ViV TAVI with BVF via transcarotid approach in a Mitroflow 19 mm valve, complicated by acute myocardial stunning and successfully rescued with emergent VA-ECMO.

## CASE REPORT

An 86-year-old man with prior AVR (Mitroflow 19 mm) and CABG using an in situ left internal thoracic artery left internal mammary artery-to-left anterior descending and right internal mammary artery-to-first marginal “Y” graft was admitted for congestive heart failure and New York Heart Association class III dyspnoea. Echocardiography showed a mean gradient of 31 mm Hg, severe prosthetic regurgitation, and preserved left ventricle ejection fraction (LVEF). Comorbidities included hypertension, chronic kidney disease, chronic obstructive pulmonary disease, peripheral artery disease, and atrial fibrillation. Computed tomography revealed a small annulus (16 × 15 mm), low coronary heights (11.8 and 8.6 mm), and heavily calcified iliofemoral arteries, with external iliac diameters of 4.4 and 4.5 mm. The left common carotid artery (LCCA) measured 6.1 mm in diameter, without calcification or tortuosity.

**Figure 1.**
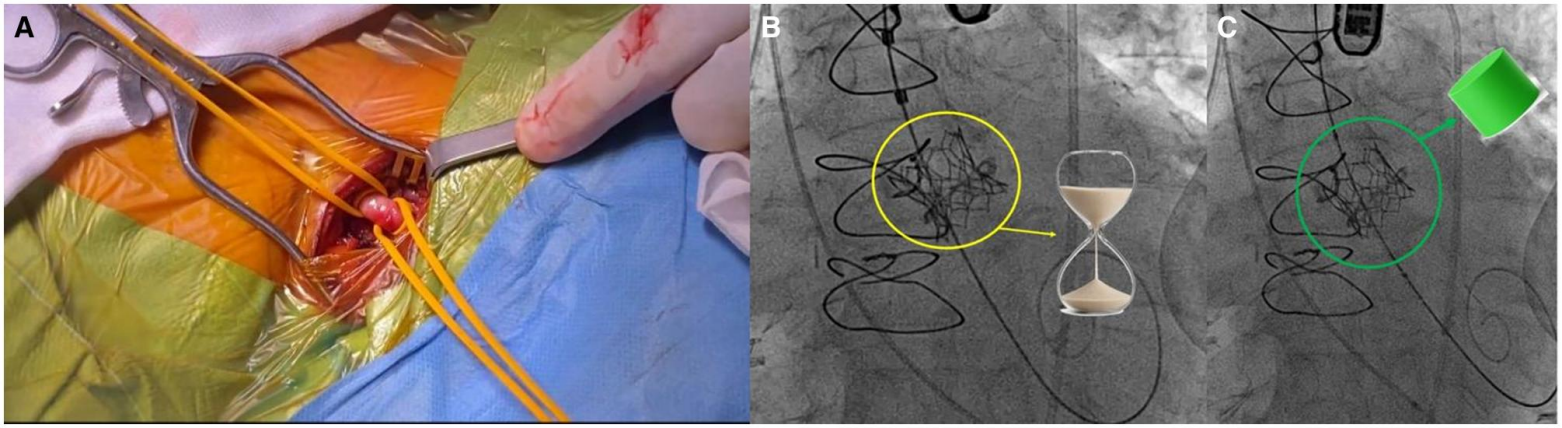
(A) Surgical exposure of the left common carotid artery for transcarotid access. (B) Fluoroscopic image showing “hourglass” waist of an underexpanded THV within a small surgical valve, indicating need for BVF. (C) Fluoroscopic image showing full expansion of the transcatheter heart valve following successful bioprosthetic valve fracture

Given the prohibitive surgical risk and unsuitable transfemoral anatomy, a transcarotid ViV procedure was planned. Under general anaesthesia and cerebral neuromonitoring, direct surgical access to the LCCA was achieved (**Figure 1A**). A 20 mm Myval transcatheter heart valve (THV) (Meril Life) was deployed inside the Mitroflow bioprosthesis. Post-deployment, fluoroscopy revealed a characteristic “hourglass” waist, indicating underexpansion (**Figure 1B** and **Video S1**). Haemodynamic assessment showed a peak velocity of 338 cm/s, peak gradient of 45.7 mm Hg, and mean gradient of 24.2 mm Hg.

**Figure 2.**
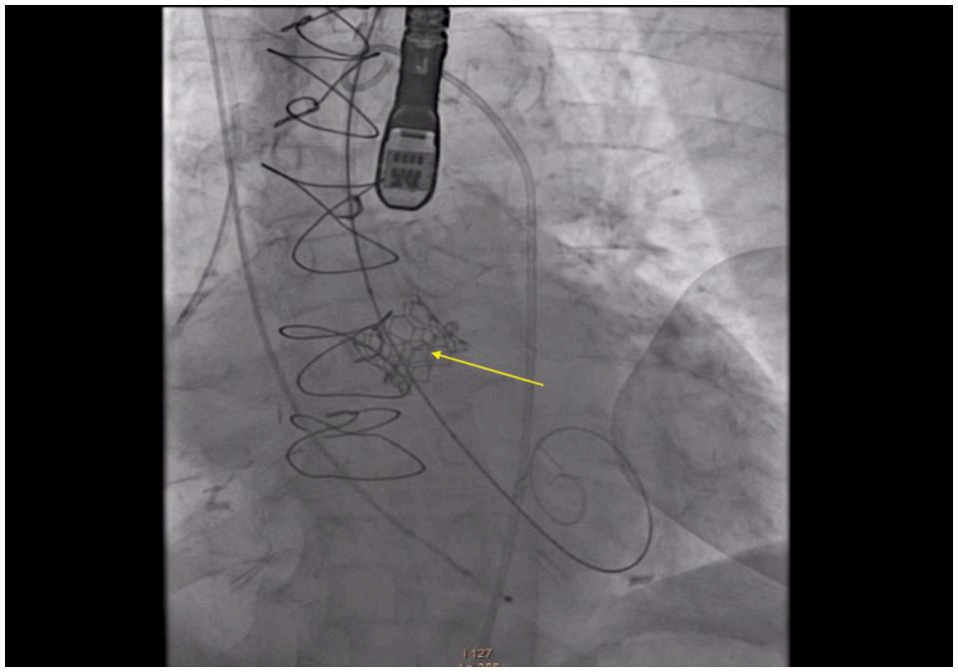
Ring Fracture Point After Balloon Valve Fracture of Mitroflow 19 mm

An 18 mm True Balloon (Bard Medical), a high-pressure non-compliant balloon, was used to perform the BVF, inflated to a nominal pressure of 22 atmospheres. Successful fracture of the surgical valve ring (**Figure 2**) was confirmed by a sudden drop in indeflator pressure and release of the balloon waist (**Figure 1C** and **Video S2**). The mean transvalvular gradient decreased from 24.2 to 11.4 mm Hg.

Shortly after balloon inflation, the patient developed severe hypotension. Transoesophageal echocardiography ruled out pericardial effusion or tamponade, and angiography confirmed patency of the coronary arteries without dissection. Acute myocardial stunning due to afterload mismatch was suspected. Emergent VA-ECMO was established via surgical cannulation of the femoral vein and external iliac artery, resulting in prompt haemodynamic stabilization (**Video S3**).

The patient was weaned from extracorporeal membrane oxygenation (ECMO) after 48 hours without neurological or vascular complications. Echocardiography at discharge showed a fully expanded valve, mean gradient of 15 mm Hg, and trivial regurgitation. He was discharged home on postoperative day 9 in NYHA class I-II. At 3-month follow-up, he remained stable and asymptomatic.

## DISCUSSION

This case illustrates the technical and haemodynamic challenges of ViV TAVI in a 19 mm Mitroflow bioprosthesis, where the narrow internal diameter and externally mounted leaflets significantly increase the risk of underexpansion and coronary obstruction. In our patient, BVF using a high-pressure non-compliant balloon enabled full THV expansion and a marked improvement in haemodynamic performance.^1–3^ The strategy used, implanting the THV first, followed by BVF, aligns with the current dominant approach described in large multicentre studies and systematic reviews.^1–3^ This sequence is favoured because it minimizes the risk of annular rupture in fragile prostheses, ensures accurate THV positioning before ring destabilization, prevents surgical ring embolization, and provides consistent radiographic and haemodynamic end-points. Therefore, the approach adopted in this case is supported by best practice and robust published evidence, particularly relevant in high-risk valves such as Mitroflow.

In our patient, BVF with a high-pressure 18 mm non-compliant balloon resulted in successful ring fracture, expansion of the underdeployed Myval THV, and significant improvement in transvalvular gradients. Shortly after balloon inflation, the patient developed hypotension. Imaging excluded tamponade or coronary obstruction. Transoesophageal echocardiography revealed transient hypokinesis of the inferior wall, suggesting myocardial stunning. Potential mechanisms include prolonged rapid pacing during balloon inflation, transient dynamic coronary compromise, or acute afterload mismatch—all of which are recognized contributors in this setting. The rapid recovery under VA-ECMO supports a reversible myocardial dysfunction.

While transfemoral access remains standard, transcarotid TAVI has emerged as the preferred non-transfemoral route due to its favourable neurological profile and growing adoption in high-volume centers.^4^ In our case, transaxillary access was contraindicated by prior CABG with in situ LIMA to LAD, posing a risk to graft perfusion. Intrathoracic options such as transapical access have been increasingly surpassed by less invasive extrathoracic approaches. The transcarotid route offered a direct and stable path with a low rate of perioperative complications.^4^

Although the iliofemoral anatomy was unsuitable for large-bore TAVI introducers, emergent VA-ECMO was feasible using smaller cannulas (15 Fr arterial and 21 Fr venous). Surgical exposure of the external iliac artery allowed safe bail-out cannulation, as aligned with current clinical practice.

To our knowledge, this is the first reported case of BVF after transcarotid ViV TAVI in a Mitroflow 19 mm valve. Emergent VA-ECMO following BVF is exceptionally rare; in a recent series, ECMO was required in ViV-TAVI cases with haemodynamic collapse, but none involved BVF.^5^ This case reinforces the need for ECMO standby in extreme-risk ViV cases involving small, rigid surgical valves and BVF.

The Myval THV offered key advantages: low profile, coaxial delivery suited for carotid access, and appropriate sizing for small annuli, making it a strong option in complex ViV scenarios.

## Supplementary Material

ivaf298_Supplementary_Data

## Data Availability

All data underlying this article are included within the manuscript. Further details are available from the corresponding author upon reasonable request.
